# Antibiofilm Effects of Oleuropein against *Staphylococcus aureus*: An In Vitro Study

**DOI:** 10.3390/foods12234301

**Published:** 2023-11-28

**Authors:** Weiping Guo, Yunfeng Xu, Yangyang Yang, Jinle Xiang, Junliang Chen, Denglin Luo, Qinggang Xie

**Affiliations:** 1 College of Food and Bioengineering, Henan University of Science and Technology, Luoyang 471023, China; guoweiping2021@163.com (W.G.); xuyunfeng@haust.edu.cn (Y.X.); 15611603163@163.com (Y.Y.); xjl5013@haust.edu.cn (J.X.); junliangchen@126.com (J.C.); 2 Heilongjiang Feihe Dairy Co., Ltd., Beijing 100015, China

**Keywords:** *S. aureus*, oleuropein, antibiofilm, extracellular polymeric substances, hydrophobicity

## Abstract

*Staphylococcus aureus* has posed a huge threat to human health and the economy. Oleuropein has antibacterial activities against various microorganisms but research on its effect on the *S. aureus* biofilm is limited. This research aimed to estimate the antibiofilm activities of oleuropein against *S. aureus*. The results suggest that the minimum inhibitory concentration of oleuropein against *S. aureus* ATCC 25923 was 3 mg/mL. The biomass of biofilms formed on the microplates and coverslips and the viability of bacteria were significantly reduced after the treatment with oleuropein. The scanning electron microscopy observation results indicated that the stacking thickness and density of the biofilm decreased when *S. aureus* was exposed to oleuropein. It had a bactericidal effect on biofilm bacteria and removed polysaccharides and proteins from mature biofilms. The effects of oleuropein on the biofilm could be explained by a reduction in bacterial secretion of extracellular polymeric substances and a change in bacterial surface hydrophobicity. Based on the above findings, oleuropein has the potential to be used against food pollution caused by *S. aureus* biofilms.

## 1. Introduction

The presence of biofilms is considered to be a key factor in food microbial contamination [1]. Extracellular polymeric substances (EPS) form the main matrix of biofilms, and their main components are polysaccharide intracellular adhesin (PIA), proteins, eDNA, and teichoic acids [2]. Biofilms protect bacteria from a variety of stress conditions, including antimicrobial agents, mechanical forces, free radicals, and host phagocytosis. In addition, microcolonies dissociate from the initial biofilm colony and adhere to the unplanted area, to encourage the growth of new biofilms [3]. Therefore, incomplete cleaning and disinfection of food processing equipment have become potential sources of microbial contamination, even leading to large-scale food poisoning incidents [4]. More worryingly, drug resistance in biofilm bacteria could be promoted by the acquisition of DNA from bacterial communities and the environment [2]. Jamali et al. analyzed 2650 samples of raw milk and dairy products, 12.4% of which were contaminated by *S. aureus*, while 16.2% were multidrug-resistant bacteria [5]. The problem of food pollution caused by biofilms of *S. aureus* needs to be solved urgently, with natural preservatives gradually attracting the attention of researchers due to their unique advantages.

Oleuropein is a phenolic secoiridoid glycoside, which consists of elenolic acid, hydroxytyrosol, and glucose molecules, and mainly exists in the *Oleaceae*. Olive leaves with oleuropein as the main bioactive component have traditionally been used in the treatment of urinary tract infections, gastrointestinal diseases, and bronchial asthma. In recent research, oleuropein was confirmed to have multiple biological activities, such as antitumor [6], antiviral [7], antidepressant [8], and cardioprotective [9] effects. In addition, previous research has recorded that oleuropein exhibited antibacterial impacts on various microorganisms. For example, Bisignano et al. measured the antibacterial activity of oleuropein against standard strains and clinically isolated strains, and the results showed that the minimum inhibitory concentrations (MICs) of oleuropein against standard strains, such as *Salmonella* Typhimurium ATCC 6539, *S. aureus* ATCC 25923, were 62.5–500 μg/mL; meanwhile, the MIC values of clinically isolated bacteria, such as *Vibrio cholerae* and penicillin-resistant *S. aureus*, were 31.25–250 μg/mL [10]. Edziri et al. reported that an extract from an olive leaf posed antibiofilm effects on *S. aureus*, *Bacillus cereus*, *Pseudomonas aeruginosa*, etc. [11]. However, the document on the antibiofilm effects of the main bioactive component in olive leaves (oleuropein) against *S. aureus* is limited. Therefore, the purpose of this research was to estimate the effect of oleuropein on the *S. aureus* biofilm and provide a new idea for the prevention and control of *S. aureus.*

## 2. Materials and Methods

### 2.1. Strains

*Staphylococcus aureus* ATCC 25923 was stored at −80 °C in tryptone soybean broth (TSB; Land Bridge Technology Co., Ltd., Beijing, China) containing 25% glycerol. It was streaked onto tryptone soya agar (TSA; Land Bridge Technology Co., Ltd., Beijing, China). The single colony was picked into TSB and cultured at 37 °C for 8 h. The optical density (OD) value of the bacterial suspension at 600 nm was adjusted to 0.5 (OD_600 nm_ = 0.5, about 10^8^ CFUs/mL) using a spectrophotometer (V-1000, Aoyi, Shanghai, China).

### 2.2. MIC of Oleuropein against S. aureus

The MIC assay was performed using the agar dilution method depicted by Qian et al. and slightly modified [12]. TSA containing different concentrations of oleuropein (≥70%, CAS 32619-42-4, Zelang Biotechnology Co., Ltd., Xi’an, China) was fully mixed and cooled to solidification. Two microliters of diluted bacterial suspension (1% of the above bacterial suspension containing about 10^6^ CFUs/mL) were added to the surface of each solid culture medium. TSA containing 1% DMSO was used as the control group. All samples were transferred to 37 °C and incubated invertedly for 24 h after the bacterial solution was dried. The minimum dilution concentration without bacterial growth was considered as the MIC.

### 2.3. Effect on the Formation of Biofilm

#### 2.3.1. Crystal Violet Dyeing Assay

The bacterial suspensions with corresponding concentrations of oleuropein (0, 1/16, 1/8, 1/4, 1/2, and 1 MIC) were added to 96-well plates, with each well containing 200 μL of the suspension. The sterile culture solution containing oleuropein at the corresponding concentration was regarded as the negative control. The 96-well plates were placed at 37 °C for 24 h, and the absorbance was determined at 630 nm by a microplate reader (Infinite E Plex, Tecan, Shanghai, China). The plates were rinsed three times with phosphate-buffered saline (PBS) and dried. A total of 250 μL of 1% crystal violet solution (Kemio Chemical Reagent Co., Ltd., Tianjin, China) was added to each well and left to stain at 37 °C for 5 min. The dye was removed, and each well was gently rinsed three times with sterile water. Then, 250 μL of 33% acetic acid solution was added to release the stain, and after 5 min, the absorbance was determined at 570 nm. The relative biofilm formation capacity was expressed as the biofilm formation index (BFI) [13], and calculated using the following equation:

BFI = (S − SC)/(G − GC)(1)

S: the OD_570_ after staining; SC: the OD_570 (control)_ after staining; G: the OD_630_ after cultivation; GC: the OD_630 (control)_ after cultivation.

#### 2.3.2. MTT Assay

The biofilm bacterial vitality was determined based on the MTT assay [14]. Each 200 μL of bacterial suspension with corresponding concentrations of oleuropein (0, 1/16, 1/8, 1/4, 1/2, and 1 MIC) was cultured in a 96-well plate at 37 °C for 24 h. The bacterial suspensions were removed and rinsed three times with PBS. Next, 250 μL of 3-[4, 5-dimethylthiazol-2-yl]-2, 5-diphenyltetrazolium bromide (MTT; 0.5 mg/mL; Sangon Biotech Co., Ltd., Shanghai, China) was added to each well and incubated at 37 °C for 3 h. Then, the MTT solution was removed and 250 μL DMSO solution was added to each well and fully dissolved. The absorbance was measured at a wavelength of 570 nm.

#### 2.3.3. Microscopic Observation of Biofilm Morphology

The biofilms were stained by crystal violet, as mentioned above. The biofilms on the coverslips were rinsed gently with PBS and the biofilm morphology was observed using a light microscope at 400× magnification after each sample had dried. The obtained images were analyzed using the software Image J 1.8.0 (National Institutes of Health, Bethesda, MD, America) to measure the coverage rate of the biofilm on the coverslips [1].

#### 2.3.4. Observation of Biofilm Morphology by Scanning Electron Microscopy (SEM)

The SEM assay was performed as described previously in Li et al., with slight modifications [15]. Briefly, stainless steel sheets were placed in TSB containing bacterial suspension (10^6^ CFUs/mL) and different concentrations of oleuropein. Then, they were cultured at 37 °C for 24 h. Biofilms attached to stainless steel sheets were rinsed gently in PBS three times and fixed overnight at 4 °C in 2.5% glutaraldehyde (Solarbio Biotechnology Co., Ltd., Beijing, China). All samples were dehydrated in different concentrations of alcohol (30%, 50%, 70%, 90%, and 100%) for 15 min. Finally, the dried samples were sprayed with Au–Pd under vacuum and observed by SEM (JSM-IT200, JEOL, Tokyo, Japan).

### 2.4. Removal Effect on the Mature Biofilm

#### 2.4.1. Crystal Violet Dyeing Assay

The crystal violet staining method in the biofilm removal assay was the same as that described above. Biofilms were formed in a 96-well plate at 37 °C for 24 h to mature. Each sample was exposed to different concentrations of oleuropein (0, 1/16, 1/8, 1/4, 1/2, and 1 MIC) at 37 °C for another 24 h. The removal rate was calculated using the following equation:

Removal rate (%) = 1 − (OD_570 (treatment)_/OD_570 (control)_) × 100% (2)

#### 2.4.2. Staining of Polysaccharides and Protein Components in Biofilm

The mature biofilm formed on the sterile coverslips was treated with oleuropein (0, 1/16 MIC, 1/8 MIC, 1/4 MIC, 1/2 MIC, and MIC) at 37 °C for 24 h. Then, the biofilm was stained in fluorescein isothiocyanate (FITC; Solarbio Science and Technology Co., Ltd., Beijing, China) [16] and fluorescent brightener 28 (FB 28; CAS: 4193-55-9; Macklin Biochemical Technology Co., Ltd., Shanghai, China) [17] for 30 min in a dark room after being rinsed with PBS three times. All samples were observed using a fluorescence microscope (DM2500, Leica, Wetzlar, Germany).

#### 2.4.3. Stained Dead Bacteria in Biofilm

As previously reported by Tremblay et al. [18], the mature biofilm was formed on sterile coverslips before the samples were exposed to corresponding concentrations of oleuropein (0, 1/2, 1, and 2 MIC) at 37 °C for 24 h. The planktonic cells were removed by washing three times with PBS. Each sample was stained in propidium iodide solute (PI; Solarbio Science and Technology Co., Ltd., Beijing, China) in the dark for 30 min. Subsequently, the stained dead cells in the biofilms were observed using a fluorescence microscope.

#### 2.4.4. Colony Counting

The count of viable biofilm bacteria was carried out according to Amalaradjou and Venkitanarayanan [19], with some modifications. The mature biofilms were formed in the wells of 6-well plates. The planktonic cells were removed with PBS by rinsing three times, and then the biofilm was treated with oleuropein (1/2 MIC, 1 MIC, and 2 MIC) and cultured at 37 °C for 2 h, 4 h, and 24 h, respectively. Then, each well was washed with PBS three times and resuspended with 3 mL PBS. A total of 100 μL of the diluted bacterial suspension was spread on TSA and cultured at 37 °C for 24 h.

### 2.5. Determination of EPS

Determination of the EPS matrix or slime was carried out according to the Congo red agar method, as described by Dos Santos et al., although with a slight modification [2]. Briefly, Congo red was combined with BHI agar medium (Land Bridge Technology Co., Ltd., Beijing, China) containing different concentrations of oleuropein (0, 1/16, 1/8, 1/4, 1/2, and 1 MIC) and cooled to solidification. Then, 2 μL bacterial suspension was added to each plate. The colony morphology was observed after culturing at 37 °C for 24 h.

### 2.6. Effect of Oleuropein on Bacterial Surface Hydrophobicity

Determination of the bacterial surface hydrophobicity was referred to for the MATH method, previously used by Tang et al., with slight modifications [20]. Briefly, the overnight *S. aureus* culture was washed and resuspended in PBS. Then, the suspensions were inoculated in TSB containing corresponding concentrations of oleuropein (0, 1/16, 1/8, 1/4, 1/2, and 1 MIC). A total of 1 mL of hexane was added to 1.5 mL of the suspension and fully mixed for 1 min followed by incubation at 37 °C for 15 min. The water layer below was extracted, and the absorbance was determined at 600 nm. The cell surface hydrophobicity was calculated using the following equation:

Hydrophobic rate (%) = (A_1_ − A_2_)/A_1_ × 100% (3)

where A_1_ is the initial absorbance at 600 nm and A_2_ is the absorbance value of each group of samples after treatment.

### 2.7. Fourier-Transform Infrared (FTIR) Spectroscopy

The effect of oleuropein treatment on the EPS of *S. aureus* was analyzed using ATR–FTIR [21]. The bacteria were washed with PBS and resuspended, with the bacterial suspensions exposed to the concentrations of 1/2 MIC and 1 MIC of oleuropein at 100 rpm, 37 °C for 8 h. The mixture was centrifuged at 10,000 rpm, 4 °C for 5 min and washed with PBS three times after cultivation. The bacterial precipitates were collected. Then, the freeze-dried powder was formed using a lyophilizer. The infrared spectra were scanned and analyzed using a Fourier-transform infrared spectrometer (VERTEX70, Bruker, Karlsruher, Germany) with a resolution of 4 cm^−1^ in the wavelength range of 400–4000 cm^−1^.

### 2.8. Statistical Analysis

Three parallel experiments were conducted in each group to obtain the mean values and standard deviations. All data were statistically analyzed using SPSS 20.0 software, and the significant differences (*p* < 0.05) between the control and treatment groups were calculated by Tukey’s multiple range test.

## 3. Results

### 3.1. MIC of Oleuropein against S. aureus

The MIC of oleuropein against the standard strain of *S. aureus* ATCC 25923 was 3 mg/mL, as determined by the agar dilution method.

### 3.2. Effect on the Formation of Biofilms

#### 3.2.1. Crystal Violet Dyeing Assay

**Figure 1:**
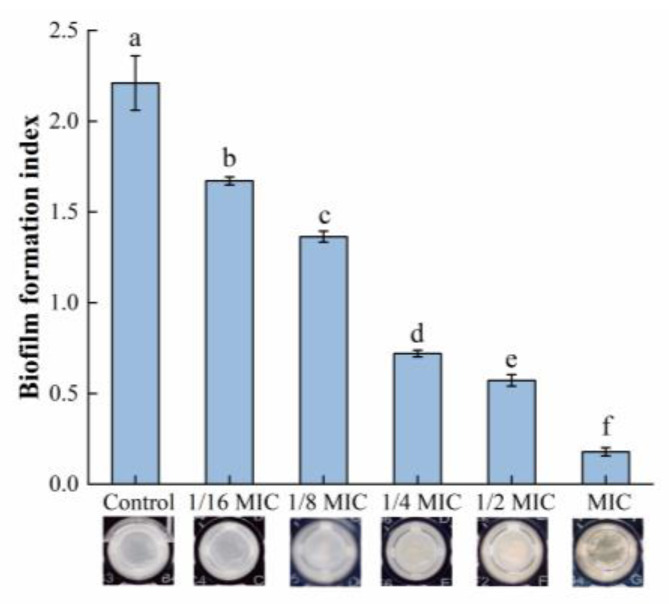
The effect of oleuropein at different concentrations on biofilm formation of *S. aureus* as detected by crystal violet staining assay. Error bars with different letters indicate significant differences (*p* < 0.05).

The relative biofilm formation in each group is shown in Figure 1. The biomass of the biofilm was significantly reduced after treatment with oleuropein within the concentration range of 1/16 to 1 MIC, and the amount of biofilm formation was reduced by 24.40% and 91.95% compared with the control group, after *S. aureus* was exposed to oleuropein at the concentrations of 1/16 MIC and 1 MIC, respectively.

#### 3.2.2. MTT Assay

**Figure 2:**
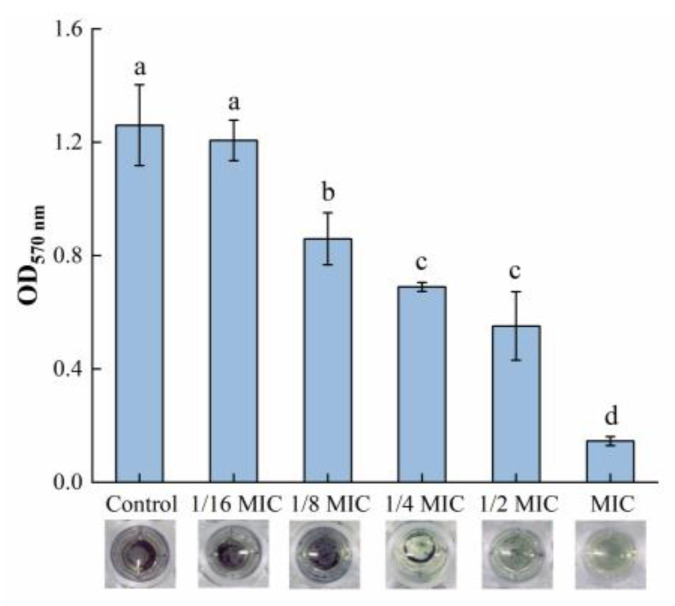
The effect of oleuropein at different concentrations on the vitality of the biofilm bacteria as measured by MTT assay. Error bars with different letters indicate significant differences (*p* < 0.05).

The MTT assay was used in this study to measure the viability of biofilm bacteria, while the result can be seen in Figure 2. Within the range of 1/8 MIC to 1 MIC, oleuropein posed a good inhibitory activity on the viability of the biofilm bacteria. The difference between the 1/16 MIC and control groups was not statistically significant (*p* = 0.59). At the highest concentration of oleuropein tested (MIC), the viability of the biofilm bacteria decreased by 88.45% compared with the control.

#### 3.2.3. Microscopic Observation of Biofilm Morphology

**Figure 3:**
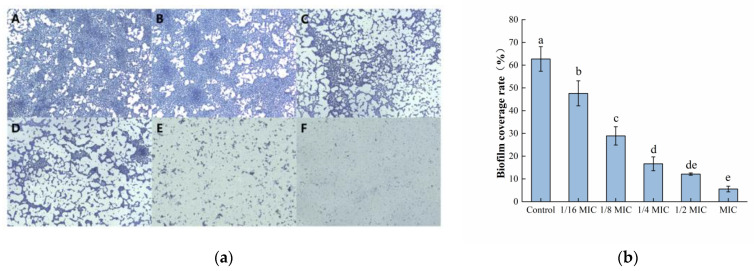
The effect of oleuropein on biofilm formation of *S. aureus*. (**a**) Observation of biofilm stained by crystal violet under optical microscope (400× magnification; the biofilm treated with oleuropein under 0 (A), 1/16 MIC (B), 1/8 MIC (C), 1/4 MIC (D), 1/2 MIC (E) and 1 MIC (F)); (**b**) The biofilm coverage rate was quantified using Image J software. Error bars with different letters indicate significant differences (*p* < 0.05).

Microscopic observations of biofilm morphology are shown in Figure 3a. The biofilm of the control group was dense and intact. However, as the concentration of oleuropein increased, the biofilm formed by bacteria gradually showed fragmentation and a downward trend. After software analysis, the amount of biofilm formation after MIC concentration of oleuropein treatment was approximately 8.87% of the control group’s (Figure 3b). The result was consistent with the crystal violet staining assay in determining the amount of biofilm formation in the above experiment.

#### 3.2.4. Observation of Biofilm Morphology by SEM

**Figure 4:**
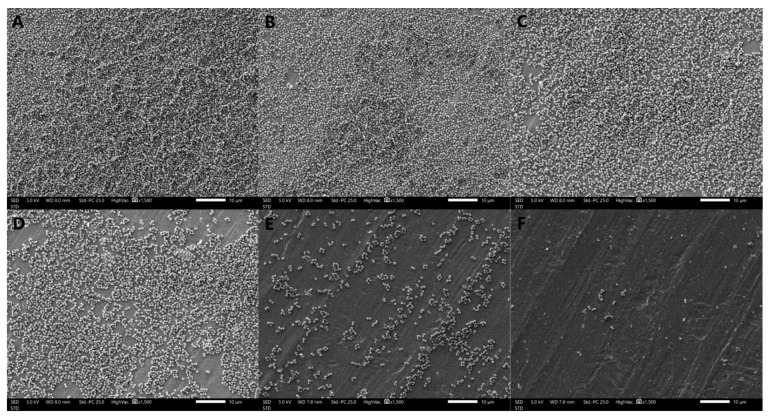
Scanning electron microscope images (1500× magnification) of *S. aureus* biofilms with oleuropein treatments of under 0 (**A**), 1/16 MIC (**B**), 1/8 MIC (**C**), 1/4 MIC (**D**), 1/2 MIC (**E**), and 1 MIC (**F**).

The formation of *S. aureus* biofilms treated with different concentrations of oleuropein observed by SEM are shown in Figure 4. The bacteria in the control group were tightly covered on the stainless-steel sheet and presented in multiple layers of stacking, indicating that the strain had strong biofilm-forming ability. As the concentrations of oleuropein increased in the treatment groups, the bacterial density and stacking layers decreased. The above results indicate that the biofilm-forming ability of *S. aureus* was significantly inhibited by oleuropein in a dose-dependent manner.

### 3.3. Removal Effect on the Mature Biofilms

#### 3.3.1. Crystal Violet Dyeing Assay

**Figure 5:**
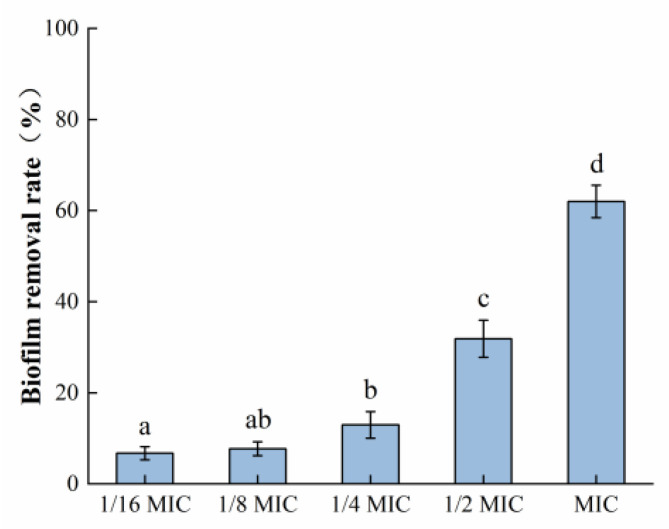
The removal effect of oleuropein at different concentrations on the mature biofilm measured using the crystal violet staining assay. Error bars with different letters indicate significant differences (*p* < 0.05).

It can be seen in Figure 5 that the mature biofilm formed by *S. aureus* was 62.02% cleared after exposure to the highest concentration of oleuropein (MIC) used in this experiment for 24 h. The above results indicated that oleuropein treatment could not only prevent the formation of *S. aureus* biofilm formation but also remove its mature biofilm.

#### 3.3.2. Staining of Protein and Polysaccharide Components in EPS

**Figure 6:**
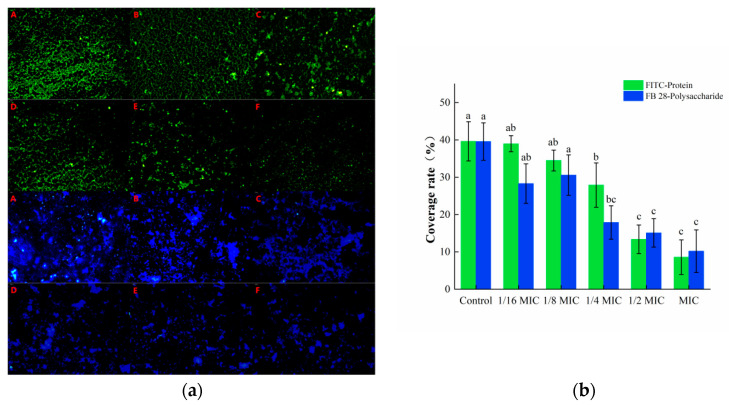
(**a**) Fluorescence microscopy photos of proteins and polysaccharides stained in mature *S. aureus* biofilms treated with different concentrations of oleuropein. (200× magnification; the biofilm treated with oleuropein under 0 (A), 1/16 MIC (B), 1/8 MIC (C), 1/4 MIC (D), 1/2 MIC (E), and 1 MIC (F)). The green signal represents protein, while the blue signal represents polysaccharide. (**b**) The green and blue signal coverage rates were quantified using the Image J software. Error bars in the same staining group with different letters indicate significant differences (*p* < 0.05).

The isothiocyanate group in the fluorescent dye FITC can bind to the free amino group in proteins, and thus, emit green fluorescence, while the FB 28 can bind to polysaccharides and emit blue fluorescence. These two substances can recognize the protein and polysaccharide components in biofilms, respectively. As shown in Figure 6a, a large amount of green and blue fluorescence was observed in the control group, indicating that *S. aureus* could secrete a number of proteins and polysaccharides required to form a biofilm. The green and blue fluorescence in the oleuropein treatment groups were markedly decreased and gradually dispersed in distribution. The software analysis results showed that after treatment with 1 MIC of oleuropein, the green fluorescence and blue fluorescence decreased by 66.29% and 54.98%, respectively, compared with the control group (Figure 6b).

#### 3.3.3. Stained Dead Bacteria in Biofilm

**Figure 7:**
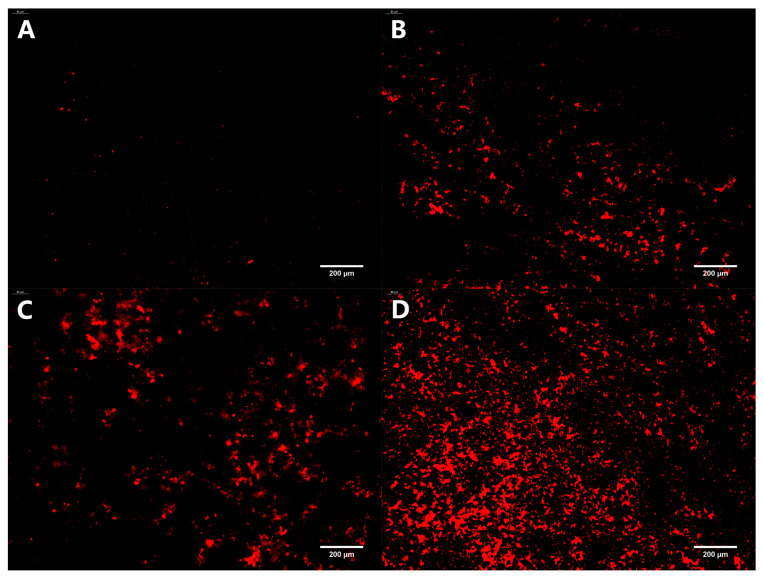
Fluorescence microscopy photos of dead bacteria stained in mature *S. aureus* biofilms treated with oleuropein at the concentrations of 0 (**A**), 1/2 MIC (**B**), 1 MIC (**C**), and 2 MIC (**D**). The images are at 200× magnification.

PI can merge with dsDNA and emit red fluorescence. However, it cannot penetrate the cell membranes of living cells, only apoptotic cells, to cause red staining of the nucleus. Therefore, the PI staining assay is commonly used to identify apoptotic cells. The distribution of dead bacteria in the mature biofilm formed on the coverslips under different treatment conditions is depicted in Figure 7. After incubating with a mature biofilm for 24 h, only a small amount of red fluorescence was observed in the control group. As the concentration of oleuropein increased, the red fluorescence also gradually increased, indicating that oleuropein had an excellent bactericidal effect on bacteria in the biofilm.

#### 3.3.4. Colony Counting 

**Figure 8:**
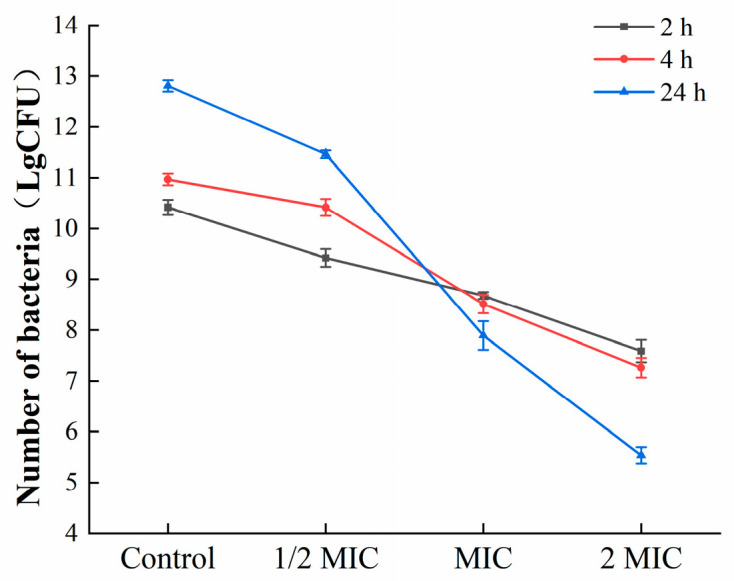
The number of viable bacteria in the mature biofilms under the treatment of oleuropein.

To further estimate the effects of oleuropein on the bacteria in the biofilm, mature biofilms were exposed to corresponding concentrations of oleuropein (0, 1/2 MIC, 1 MIC, and 2 MIC), and the colonies were counted. As shown in Figure 8, oleuropein inhibited the growth of *S. aureus* in mature biofilms in a dose-dependent manner.

### 3.4. EPS Assay

**Figure 9:**
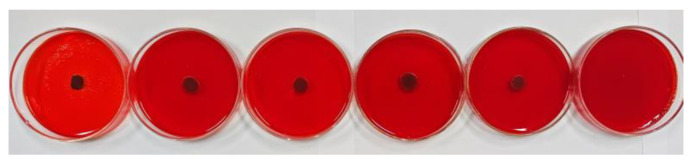
The level of EPS production by *S. aureus* under the treatment of oleuropein at concentrations of 0, 1/16 MIC, 1/8 MIC, 1/4 MIC, 1/2 MIC, and 1 MIC, in sequence.

The Congo red method was used to further evaluate the antibiofilm activity of oleuropein, with the result depicted in Figure 9. After cultivating for 24 h, the colonies in the control group were black and had dry crystals, indicating that the bacteria had secreted EPS matrix or slime, meaning they had already formed a biofilm. As the concentration of oleuropein increased, the blackness of the colonies gradually decreased, and the surrounding crystals disappeared. The result indicated that the bacterial capacity to form a biofilm had been inhibited.

### 3.5. Determination of Bacterial Surface Hydrophobicity

**Figure 10:**
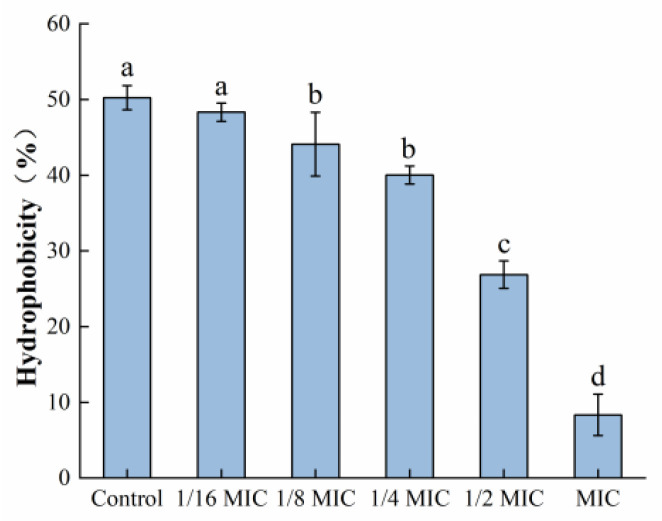
The effect of oleuropein at different concentrations on the hydrophobicity of *S. aureus.* Error bars with different letters indicate significant differences (*p* < 0.05).

As depicted in Figure 10, the surface hydrophobicity of *S. aureus* was decreased after treatment with oleuropein within the concentration range of 1/6 MIC to 1 MIC, in a dose-dependent manner. At the MIC concentration, it had decreased by 87.44% compared to the control group.

### 3.6. Fourier-Transform Infrared (FTIR) Spectroscopy

**Figure 11:**
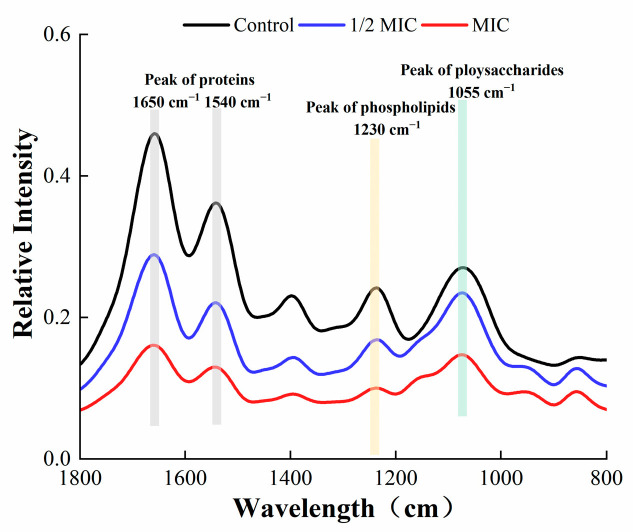
Fourier-transform infrared spectra of *S. aureus* biofilm within the wavelength range of 800–1800 cm^−1^.

The FTIR spectroscopy of *S. aureus* in the wavelength range of 800–1800 cm^−1^ is shown in Figure 11. The content of proteins, phospholipids, and polysaccharides in the EPS of *S. aureus* was markedly reduced after treatment with oleuropein. Such substances are the main component of EPS produced by bacteria, and a decrease in secretion was not conducive to the formation of the bacterial biofilm.

## 4. Discussion

The formation of biofilms is considered a key virulence factor in pathogenesis, which helps colonies to survive under extreme conditions and enhance their resistance to invasive antibiotics and host immune responses. Given the crucial role of biofilms in bacterial antibiotic resistance, it is necessary to explore new alternative sources that target biofilm formation in bacterial pathogens. Due to breakthroughs in the exploration of folk medicinal plants for the treatment of human diseases, people tried to investigate the effect of folk medicinal plants on inhibiting the formation of biofilms by bacterial and fungal pathogens and the production of related virulence factors [21]. *S. aureus* has strong biofilm-forming abilities and poses a huge threat to the food industry [22]. This study mainly investigated the antibiofilm effects of oleuropein against *S. aureus*. Oleuropein at concentrations of 1/16 MIC to 1 MIC could significantly reduce the production of biofilm biomass and remove polysaccharides and proteins from mature biofilms at different levels. Many phytocompounds have been confirmed to exhibit similar effects on bacterial biofilms. For instance, carvacrol reduced the metabolic activity of *Streptococcus* in mature biofilms [23]. The biofilm formation of *Yersinia enterocolitica* was inhibited when exposed to chlorogenic acid, by disrupting the physiological pathways of biofilm formation [24]. The essential oil extracted from different varieties of *Lippia alba* by hydrodistillation could 100% inhibit the biofilm formation of *S. aureus* when the concentration reached 0.5 mg/mL, and the cells in the biofilms were removed within the range of 1 to 2 mg/mL [25]. As for the commonly used medicinal plants that resist *Staphylococcus* infection, the crude extracts of *Alnus japonica* [26], *Rubus ulmifolius* [27], and *Quercus cerris* [28] have been documented to inhibit the formation of the *Staphylococcus* biofilm.

Microscopic morphology of the biofilm formed by *S. aureus* under oleuropein treatment was observed by SEM. The biofilm formed by *S. aureus* in the control group was dense and stacked in multiple layers. However, as the concentration of oleuropein in the environment increased, the density of the biofilm decreased. Similarly, some natural compounds have been confirmed to pose a destructive effect on the structure of biofilms produced by bacteria. The extract of marine sponge effectively reduced the bacterial community in the *Candida* species biofilm, changed the morphology of the biofilm, and destroyed the mature biofilm [29]. Treatment with resveratrol at 32 μg/mL in combination with florfenicol at 64 μg/mL could also eradicate the biofilm formed by avian pathogenic *Escherichia coli* [30]. Sisto et al. observed that the biofilm structure formed by clinical strains of *Helicobacter pylori* was changed after the treatment with both the arylaminoartemisinin GC012 and dihydroartemisinin [31].

EPS play a crucial role in maintaining the structural integrity of biofilms [32]. They are conducive to bacteria anchoring the host [33], and encapsulate microorganisms to resist adverse environments [34]. In this study, FITR and the Congo red assay were used to detect the secretion of EPS by *S. aureus.* As described by previous research, the infrared spectral characteristic peaks of 1650 and 1540 cm^−1^ represent amide bonds in proteins, the peak at 1230 cm^−1^ represents phospholipids, and the peak at 1055 cm^−1^ represents polysaccharides [35]. Our results showed that the spectral characteristic area of proteins, phospholipids, and polysaccharides decreased, indicating that the secretion of EPS was affected, which was confirmed by the Congo red test. The fluorescein staining was used to estimate the changes in protein and polysaccharide components in mature biofilms after oleuropein treatment. Both of the components were effectively removed after treatment with oleuropein at 1/4 MIC to 1 MIC. A study conducted by Packiavathy et al. showed a decrease in EPS production by *Vibrio* after treatment with curcumin [36].

The four stages of bacterial biofilm formation are attachment, growth, maturation, and diffusion. The attachment stage is crucial for the formation of bacterial biofilms, and the surface hydrophobicity of bacteria is closely related to their adhesion ability. The stronger the surface hydrophobicity of bacteria, the stronger their adhesion ability [37]. In this study, the *S. aureus* surface hydrophobicity was significantly reduced after treatment with oleuropein, which may eventually result in a decreased adhesion ability and interfere with the biofilm formation. Similarly, vitexin reduced the surface hydrophobicity of *S. aureus* at a concentration of 26 μg/mL [38]. Kim et al. found that a number of fatty acids could reduce the hydrophobicity of *Cutibacterium acnes*, which is related to their ability to resist the biofilm [39].

Although oleuropein has the potential to become an antibiofilm agent against *S. aureus*, the application parameters for phytochemicals in the food industry remain under consideration. Rambabu et al. reported a composite preservative film made of mango extract and chitosan that showed good effects against food oxidation [40]. De Figueiredo et al. added *Croton blanchetianus* Baill extract to lamb ribs and effectively delayed lipid oxidation [41]. Additionally, some reports might provide references for the application of olive extract in food. Bonolive^TM^ is a patented extract from olive leaves produced in Spain, which is based on the standard of the Global Food Safety Initiative with oleuropein as the main component and has been proven to be safe in some human experiments [42,43]. Fei et al. found that olive oil polyphenol extract could reduce the number of *Bacillus cereus* in pasteurized milk [44]. The possible interactions between oleuropein and food components and oleuropein dosage optimization need to be carried out in the future.

## 5. Conclusions

In conclusion, this research confirmed that oleuropein exhibited a good ability to inhibit *S. aureus* biofilm formation and remove its mature biofilm. Oleuropein dose-dependently reduced the biomass of the biofilm formed on the microplates and coverslips, decreased the viability of bacteria, and impaired the biofilm structure. In addition, oleuropein treatment could significantly reduce the protein and polysaccharide components in mature biofilms and promote a dose-dependently bactericidal effect on the bacteria in the biofilm. The effect of oleuropein on the *S. aureus* biofilm could be partly related to reduced EPS secretion by bacteria and the altered hydrophobicity of the cell surface. Accordingly, our research suggests that oleuropein has great potential for combating food pollution caused by *S. aureus* biofilm.

## Data Availability

The data used to support the findings of this study can be made available by the corresponding author upon request.
